# Toward Better Semantic Interoperability of Data Element Repositories in Medicine: Analysis Study

**DOI:** 10.2196/60293

**Published:** 2024-09-30

**Authors:** Zhengyong Hu, Anran Wang, Yifan Duan, Jiayin Zhou, Wanfei Hu, Sizhu Wu

**Affiliations:** 1 Institute of Medical Information/Medical Library Chinese Academy of Medical Sciences & Peking Union Medical College Beijing China

**Keywords:** data element repository, FAIR, ISO/IEC 11179, metadata, semantic interoperability

## Abstract

**Background:**

Data element repositories facilitate high-quality medical data sharing by standardizing data and enhancing semantic interoperability. However, the application of repositories is confined to specific projects and institutions.

**Objective:**

This study aims to explore potential issues and promote broader application of data element repositories within the medical field by evaluating and analyzing typical repositories.

**Methods:**

Following the inclusion of 5 data element repositories through a literature review, a novel analysis framework consisting of 7 dimensions and 36 secondary indicators was constructed and used for evaluation and analysis.

**Results:**

The study’s results delineate the unique characteristics of different repositories and uncover specific issues in their construction. These issues include the absence of data reuse protocols and insufficient information regarding the application scenarios and efficacy of data elements. The repositories fully comply with only 45% (9/20) of the subprinciples for Findable and Reusable in the FAIR principle, while achieving a 90% (19/20 subprinciples) compliance rate for Accessible and 67% (10/15 subprinciples) for Interoperable.

**Conclusions:**

The recommendations proposed in this study address the issues to improve the construction and application of repositories, offering valuable insights to data managers, computer experts, and other pertinent stakeholders.

## Introduction

### Background

The sharing of medical data can enhance the efficiency of medical research, bolster transparency within the field of medicine, and respond to the stringent demands for research reproducibility and data openness [1]. Nonetheless, medical data present challenges due to their high complexity in semantics and heterogeneity, and they lack standards and uniform specifications at the level of fields and value domains. For instance, the numeric value “18” could represent the age at which a patient started smoking in one study, while in another, it might signify the total number of years a person has been smoking. This issue of semantic ambiguity in the data renders it challenging for other researchers to comprehend and use the information. It impedes the integration, comparison, and joint analysis of different data sets [2], thereby obstructing data sharing.

Metadata, essentially data about data, offers a solution to address such issues. Metadata can describe data, providing researchers with a comprehensive overview to aid understanding and application. Furthermore, it supports more precise retrieval and traceability. When data are accurately associated with metadata (such as “18” being linked to an individual’s total years of smoking), its semantics become much more straightforward. Metadata has already found applications in various fields, including molecular biology [3,4] and clinical medicine [5,6]. Guidelines for data management and sharing, such as the FAIR (Findable, Accessible, Interoperable, and Reusable) principles, also provide specifications for metadata to ensure that data are Findable, Accessible, Interoperable, and Reusable [7]. However, researchers often find that creating and annotating metadata are time-consuming and prone to errors [8]. This makes it challenging to ensure metadata quality and increases metadata heterogeneity across research. Hence, using standardized metadata for data collection to achieve semantic consistency from the inception of the data life cycle is essential to maximize semantic interoperability across multiple data sources.

Data elements (DEs) are vital components of metadata, representing indivisible data units within a given context. The underlying framework of DEs can furnish rich metadata information, including unique identifiers, definitions, and value domains, among other attributes. The DE repository represents a platform structured in accordance with a standardized framework dedicated to the construction, storage, administration, and dissemination of DEs. Within this repository, DEs adhere to rigorous standardization, with their conceptual aspects, value ranges, and related attributes systematically linked to controlled vocabularies and other terminological systems. A DE repository facilitates the unified management and maintenance of internal metadata, ensuring semantic consistency and reducing the cost associated with redundant design efforts for project-specific metadata. By fostering the reuse and sharing of standardized DEs, barriers to data integration are diminished, thus propelling applications such as cross-institutional and cross-study meta-analyses in the realm of medical data [9]. This, in turn, unlocks the value of medical data.

Currently, the prevailing international standards for DEs and repository construction are set by the ISO/IEC (International Organization for Standardization/International Electrotechnical Commission) 11179 standard. The ISO/IEC 11179 standard establishes a conceptual model for DEs and their repositories, while also providing regulations for activities such as DE registration and management. Many DE repositories have been constructed in the medical field based on the ISO/IEC 11179 standard. However, the broader application of DE repositories has not yet been achieved, often limited to specific projects or internal use within particular institutions [10]. As the central platform for storing, managing, and sharing DEs and metadata, the degree of completeness in its construction directly influences the practical usage of DEs. Current research tends to focus more on the specific technical aspects and standards for constructing DE repositories. Simultaneously, there is a discernible deficiency in evaluating and analyzing typical repositories in the medical domain.

### Literature Review

#### Medical DE

Data elements, defined and standardized by the ISO/IEC 11179 standard, constitute the minors units for collecting, processing, and disseminating data [11]. The definition of DEs should ideally encompass 3 aspects—research questions, data acquisition, and data storage—to reflect the life cycle of a repository best [12]. DEs play a pivotal role in standardizing clinical data collection, enhancing data quality, facilitating secondary analysis and applications [13,14], and serving as a base for systems based on artificial intelligence (AI) [15].

Currently, the development of Des primarily relies on multidomain expert consensus and collaboration, often achieved through iterative Delphi methods for discussion, identification, and refinement of relevant DEs [16]. This approach ensures the professionalism of DEs within specific domains but demands considerable time and personnel involvement. National Institute of Neurological Disorders and Stroke (NINDS) categorizes the development of common data elements (CDEs) into 4 phases: discovery, internal validation, external validation, and distribution [17]. Numerous domains or projects have undergone multiple iterations of DE development, such as Stroke V2.0 CDE [18]. More granular domain-specific DEs have been developed or reached consensus, spanning therapeutic methods [19], examinations [20], and others. With the continuous expansion of DEs, Kim et al [21] proposed a comprehensive representation of real-world clinical semantics by defining semantic relationships and constraints between DEs.

The application and evaluation of DEs have indeed garnered considerable attention. For instance, Fitzgerald et al [22] analyzed the seizure burden using clinical data in childhood epilepsies collected from CDE-based forms within the electronic medical record. Evaluation studies encompass DE quality [23] and the effectiveness of data collection. Chen et al [24] assessed the data collection effectiveness of DEs in real-world scenarios, while Ryan et al [25] separately evaluated data capture rates for DEs in in-person and virtual visits scenarios.

Recently, several studies have sought to advance the application of AI technologies throughout the life cycle of DEs. Natural language processing can assist in extracting specific DEs from clinical documents [26-28]. Renner et al [29] explored the use of artificial neural networks to semiautomatically map DE models to the BRIDG model, thereby reducing the burden of manual mapping by experts. In addition, DEs play a role in collecting high-quality data to aid in training machine learning algorithms, further expanding their applications in the health information domain [30]. Littlefield et al [31], based on data collected through DEs, compared the performance of major machine learning algorithms with traditional statistical methods.

#### DE Repository

DE repositories serve as platforms for storing and managing DEs, facilitating standardization, and promoting the integration and sharing of medical data through both top-down and bottom-up approaches [32]. The bottom-up approach relies on users creating and maintaining their DEs. Hegselmann et al [33] have expanded upon this model by extracting real-world DEs from medical documents and standardizing them, thereby promoting the reusability of DEs. The DE repository can standardize metadata across various studies and institutions, facilitating data integration. Mallya et al [34] coordinated variables in 4 research endeavors through the effective usage of the DE repository.

Another crucial function of the DE repository is to ensure internal semantic consistency, thereby enhancing the semantic interoperability of DEs. One perspective suggests that the maintenance and updating of terms should be separated from the repository’s operational tasks [35]. Schladetzky et al [36] developed the Mettertron system to enhance the linkage between the DE repository and the terminology system, simplifying terminology maintenance services. Meanwhile, mapping the repository model to the Web Ontology Language (OWL) ontology model can expand its semantic applications. Yuan and Li [37] constructed a semantic relation metamodel for the repository and defined mapping rules to the ontology model.

Recent research has also been conducted on data quality assessment based on the DE repository. For instance, Juárez et al [38] attempted to validate local data repositories by the central DE repository of networks, thereby providing a comparative method for assessing data quality across different sites. Kapsner et al [39] centralized the maintenance of data quality checks by associating data quality assessment tools with DE definitions in the DE repository.

#### Related Works

Current research lacks a comprehensive evaluation and analysis of multiple typical medical DE repositories. Ulrich et al [40] referenced information about specific metadata repositories in evaluating the application of the metadata exchange language QL4MDR. Hegselmann et al [33] also provided a brief overview of repository practices based on the ISO/IEC 11179 standard in his study on Pragmatic MDR. Nonetheless, both studies stopped short of providing a detailed evaluation or analysis and did not endeavor to suggest an analytical framework or standard.

Sasse et al [41] conducted a literature review on semantic annotation services for biomedical metadata. Through the review, they identified 10 supporting tools and conducted a detailed comparison based on 7 criteria. While their comparative dimensions are unidimensional and more aligned with tools rather than repositories, the variables in their semantic services provide a reference for the semantic dimensions in constructing the analytical framework for this study.

Stoehr et al [42] assessed the portal usability of the CoMetaR repository. They divided the web page into different modules and used the Think Aloud method along with a usability scale, conducting a combined quantitative and qualitative evaluation. While their method of module-based usability assessment provides insights for constructing usability evaluation dimensions in this study, it is worth noting that their focus is on optimizing the web page’s interaction and does not compare it with the web pages of other repositories. Reichenpfader et al [43] similarly assessed the usability of the Portal of Medical Data Models (MDM-Portal) repository by analyzing the users’ experience with the web page through various tests. The dimensions they analyzed also provide insights for the usability evaluation in this study.

### Objectives

The primary objective of this study is to explore potential issues and promote the broader application of DE repositories within the medical field by evaluating and analyzing typical repositories. Furthermore, we also endeavor to address the gap in the existing literature concerning the lack of evaluation of DE repositories, offering an overview of the typical DE repository construction in the medical field.

## Methods

**Figure 1:**
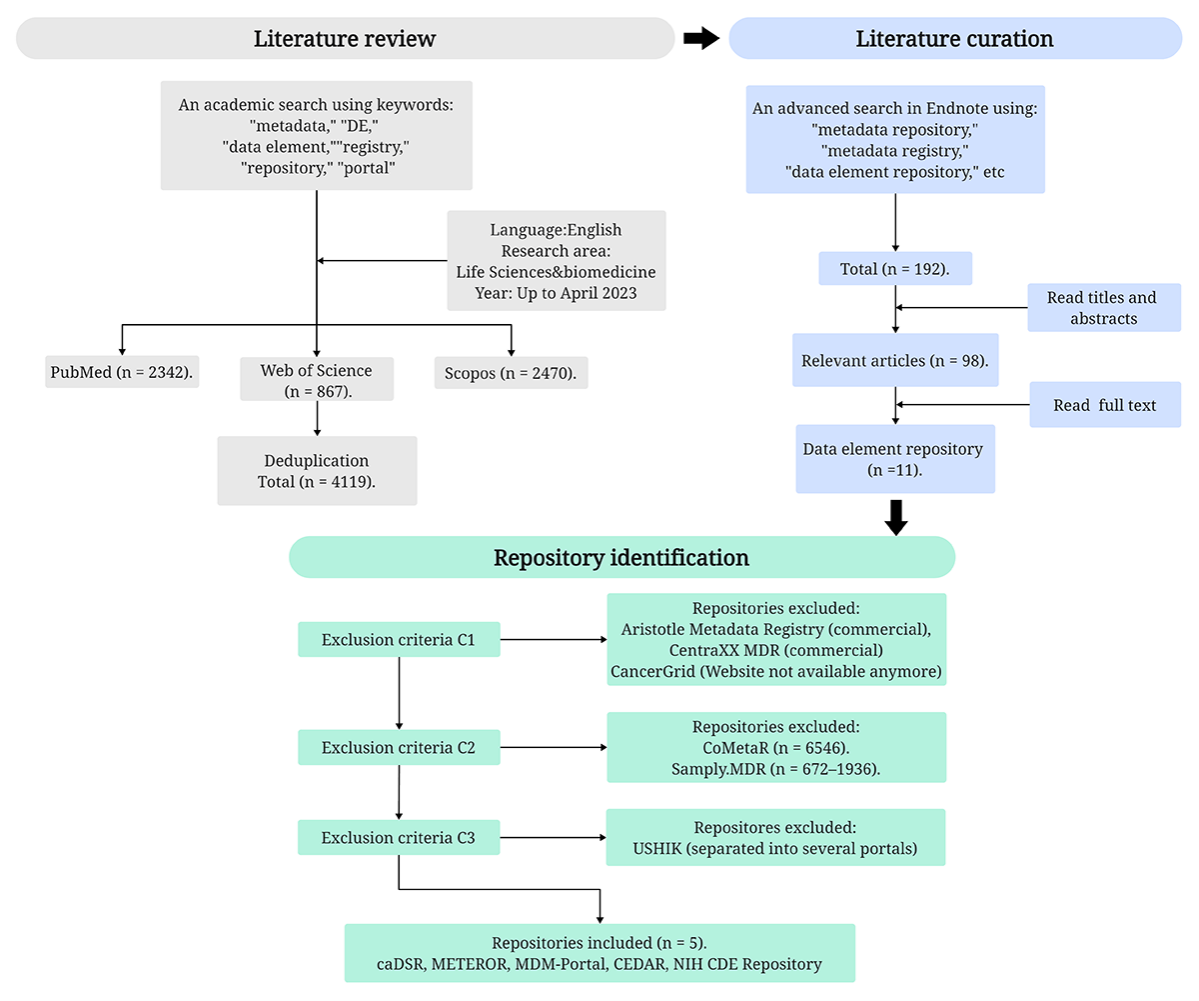
Flowchart of screening the data element repository for this study through literature review. The upper left gray part is the first step: searching literature from various databases. The upper right blue part is the second step: obtaining the data element repository through further screening and reading of literature. The green part below is the third step: obtaining the data element repository for this study according to the 3 inclusion and exclusion criteria: C1, C2, and C3. caDSR: Cancer Data Standards Registry and Repository; CDE: Common Data Element; CEDAR: Center for Expanded Data Annotation and Retrieval; DE: Data Element; MDM-Portal: Portal of Medical Data Model; METEOR: Metadata Online Registry; NIH: National Institutes of Health.

The method used in this study for screening medical DE repositories involves three distinct steps: (1) literature review, (2) literature curation, and (3) repositories identification (Figure 1).

### Literature Review

This study conducted literature searches on PubMed, Web of Science, and Scopus. The searches were performed using a combination of keywords such as “metadata,” “data element,” and “DE,” combined with “repository,” “registry,” “platform,” and “portal.” The language was restricted to English, and the research area was focused on life sciences or biomedicine. Up to April 2023, a total of 4119 papers were retrieved.

### Repository Curation

The retrieved literature was imported into Endnote, and an advanced search was conducted explicitly targeting titles or abstracts containing terms such as “metadata repository,” “metadata registry,” and “data element repository.” After this secondary screening, a total of 192 papers were obtained. After reviewing the titles and abstracts of these papers, 98 papers related to DE repositories were identified and subsequently read in full. In the end, 11 DE repositories (shown in Table 1) within the medical field were gathered. The information and data related to DE repositories were primarily collected from three sources: (1) the portals of various repositories, (2) relevant literature, and (3) project archives up to April 2023.

**Table 1:** Eleven data elements repositories retrieved from literature (repository URLs in references).

Data element repositories	Country
Samply.MDR [44]	Germany
MDM.Portal [45]	Germany
CoMetaR [46]	Germany
CentraXX MDR [47]	Germany
CancerGrid (2005-2010) [48]	United Kingdom
METEOR (METeOR) [49]	Australia
Aristotle Metadata Registry [50]	Australia
caDSR [51]	United States
USUIK [52]	United States
NIH CDE Repository [53]	United States
CEDAR [54]	United States

### Repository Identification

To facilitate a more effective comparison, we established inclusion and exclusion criteria for screening the 11 repositories. The specific inclusion and exclusion criteria and the process are as follows:

1. C1: DE repositories should be open-access public platforms, meeting noncommercial or managed by nonprofit organizations (such as universities or research institutions).
2. C2: The repository’s metadata or DE resources should comprise more than 20,000 records.
3. C3: We required the repository to have a well-established, independent portal to support access.

Five DE repositories were ultimately included (Table 2): Cancer Data Standards Registry and Repository (caDSR) [55], NIH (National Institutes of Health) CDE Repository, MDM-Portal [2], Metadata online registry (METEOR), and Center for Expanded Data Annotation and Retrieval (CEDAR) [56].

**Table 2:** Basic information of the 5 data element repositories included in this study.

Repositories	Country	First release year	Hosted by
caDSR^a^	America	2003	National Cancer Institute
NIH^b^ CDE^c^ Repository	America	2015	National Library of Medicine
METEOR^d^	Australia	2022	Australian Institute of Health and Welfare
MDM^e^	Germany	2012	Heidelberg University Hospital
CEDAR^f^	America	2014	Stanford University

^a^caDSR: Cancer Data Standards Registry and Repository.

^b^NIH: National Institutes of Health.

^c^CDE: Common Data Element.

^d^METEOR: Metadata Online Registry.

^e^MDM: Medical Data Model.

^f^CEDAR: Center for Expanded Data Annotation and Retrieval.

### Analysis Framework

We aimed to comprehensively analyze the repositories, encompassing multiple dimensions, including technology, management, and services. To achieve this, we developed a comprehensive analysis framework consisting of 7 dimensions and 36 secondary indicators (Figure 1). The 7 dimensions include the following:

1. *Data resources*: providing an overview of the repository’s data resources, including data volume, data types, data sources, coverage, and domains.
2. *Resource organization*: focusing on how metadata or DE resources are effectively organized and managed throughout their life cycle, including underlying frameworks, traceability, and version control.
3. *Quality control*: analyzing how the platform ensures the quality of stored data.
4. *Semantic annotation*: assessing how the repository achieves internal semantic consistency to enhance semantic interoperability.
5. *Service support*: examining the services offered to users by the repository, including basic services, such as retrieval and download, and advanced features such as analysis tools.
6. *Usability*: evaluating the platform’s openness, accessibility, and intelligibility, including the availability of support documents and training materials.
7. *Practice of FAIR principles*: finally, analyzing the repository’s adherence to the FAIR principles as a supplementary assessment.

**Figure 2:**
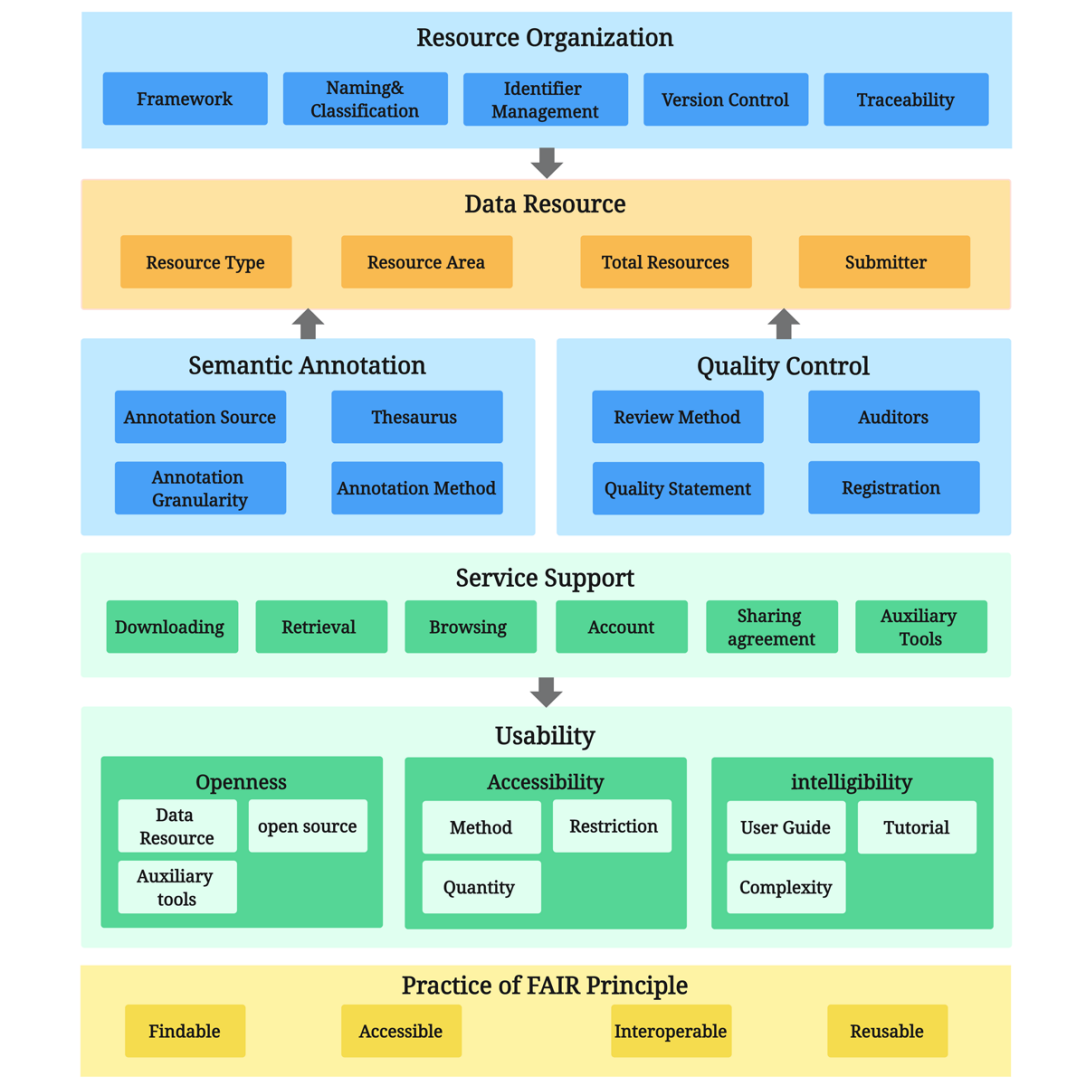
The analysis framework constructed in this study. The 7 light-colored parts represent the 7 analysis dimensions, and the dark rectangle in the middle is the specific indicator of each dimension. FAIR: Findable, Accessible, Interoperable, and Reusable.

Data resources and services dimensions are primarily determined by repository and portal characteristics, while resource organization, quality control, and semantics leverage insights from relevant literature and the ISO/IEC 11179 standard. Practice of FAIR adheres to the FAIR principle and its 15 subprinciples. Furthermore, 4 experts in data management, data warehousing construction, and data standardization participated in consultations to refine the analysis framework. Their input informed the division, naming, and selection of secondary indicators for the dimensions. The analysis framework was further refined based on expert suggestions primarily through (1) revising the name of Semantic Annotations and Service Support dimensions; (2) dividing the Usability dimension into 3 distinct modules: openness, accessibility, and intelligibility; and (3) adding more granular secondary indicators, such as source link and historical versions encoding, to enhance the depth of analysis (Figure 2). For a detailed description of the indicators included in this analytical framework, see Multimedia Appendix 1.

## Results

### Data Resource

A comparison of the data resources of the 5 repositories was conducted (Table 3). The data resources of all 5 repositories are comprehensive, but each has its emphasis on specific subdomains. For example, caDSR focuses on cancer-related DEs, METEOR emphasizes health and welfare, while others encompass DEs from various biomedical research domains. The types of resources in the repositories include elements and forms. However, the names for DEs are not yet standardized across the repositories and may consist of terms such as CDE, DE, and Field, among others. Resources in caDSR, METEOR, and NIH CDE Repository are sourced from government and institutional research projects and are released through a top-down approach. In contrast, the other platforms rely on contributions from individual users, following a bottom-up data source model. The latter category tends to have a larger volume of resources, with MDM, for instance, cataloging the most extensive collection of DEs, totaling up to 500,000 elements and more than 20,000 forms.

**Table 3:** Analysis results of the 5 data element repositories in the data resource dimension.

Repositories	Area	Type	Total amount	Submitter
caDSR^a^	Cancer research, etc	DEs^b^	71,743 DEs	NIH^c^ research institutes and programs
NIH CDE^d^ Repository	Biomedical field	DEs and forms^e^	20,970 DEs; 1704 forms	NIH research institutes and programs
METEOR^f^	Health and welfare	DEs	21,180 DEs	Australian health department or research institution
MDM^g^	Clinical trials, special diseases, etc	DEs and forms	500,000 DEs; 24,810 forms	Individual user or project submissions
CEDAR^h^	Biomedical field	DEs and forms	120,829 DEs; 2000 forms	Individual user or project submissions

^a^caDSR: Cancer Data Standards Registry and Repository.

^b^DEs: Data Elements.

^c^NIH: National Institutes of Health.

^d^CDE: Common Data Elements.

^e^Forms: forms composed of data elements (eg, case report forms, questionnaires).

^f^METEOR: Metadata Online Registry.

^g^MDM: Medical Data Model.

^h^CEDAR: Center for Expanded Data Annotation and Retrieval.

### Resource Organization

Regarding repository frameworks, all repositories except for MDM are constructed based on the ISO/IEC 11179 standard (Table 4). The DEs in these repositories are built upon the conceptual model of DEs and value domains outlined in the ISO/IEC 11179 standard. METEOR has extended this framework by introducing a top-level category called “data set specification” (DSS). This category is used to group specific DEs. For example, the “Diabetes (Clinical) DSS” in METEOR contains DEs related to standardized data collection for patients with diabetes. In MDM, which uses a custom DE framework, the attributes of DEs are relatively concise. They typically include only the DE description, data type, concept, and value domain information.

All repositories have assigned unique identifiers to their resources, although the granularity of the assignment varies. In the case of MDM, the smallest unit assigned an internal identifier is a form, and unique identifiers are not provided for individual DEs. On the other hand, other repositories assign unique identifiers at the level of individual DEs. Furthermore, the encoding of unique identifiers is standardized only within caDSR and METEOR. Simultaneously, some other repositories have inconsistent encoding methods for resources at the same hierarchical level, or they directly reference the source identifiers.

Regarding external provenance, most platforms can provide basic provenance information for DEs, such as the data submitter or the source institution. Among them, MDM provides the most detailed information, including the owner or institution of the DE, source links, and partial contact information. Regarding internal referencing and provenance, METEOR demonstrates the most comprehensive practice. It can support granularity to value domains, object classes, and properties. DEs in METEOR are listed with links to the attributes they reference and from which elements they are derived. Corresponding attributes such as value domains and object classes also provide links to all DEs that reference them. This allows for bidirectional provenance between elements and attributes.

**Table 4:** Analysis results of the 5 data element repositories in the resource organization dimension, with further explanation of identifiers, traceability, and version control indicators.

Repositories	Framework	Naming specification	Classification scheme	Identifier			Traceability						Version control	
				Name	Encoding	Submitter/source information		Source link	Source identifier	Internal citation link	Historical versions accessible			Version encoding
caDSR^a^	ISO/IEC 11179	Yes	Yes	Public ID	7 digits	Yes		No	No	No	No			Yes
NIH^b^ CDE^c^ Repository	ISO/IEC 11179	No	Yes	Identifiers	N/A^d^	Yes		No	Yes	Yes	Yes			No
METEOR^e^	ISO/IEC 11179	Yes	Yes	Identifiers	6 digits	Yes		Yes	No	Yes	Yes			No
MDM^f^	N/A	No	Yes	Public ID	N/A	Yes		Yes	No	No	Yes			Yes
CEDAR^g^	ISO/IEC 11179	No	No	Identifiers	N/A	N/A		No	Yes	No	No			No

^a^caDSR: Cancer Data Standards Registry and Repository.

^b^NIH: National Institutes of Health.

^c^CDE: Common Data Elements.

^d^N/A: not applicable.

^e^METEOR: Metadata Online Registry.

^f^MDM: Medical Data Model.

^g^CEDAR: Center for Expanded Data Annotation and Retrieval.

The version number formats for DEs in most repositories lack uniformity; in some cases, no version numbers are provided. In addition, some repositories do not allow access to historical versions of DEs, making them inaccessible for viewing. MDM has better version control practices in place. Historical versions of DEs are accessible and come with a standardized version number format. The version number includes a detailed editing data and information about the editor (eg, “4/6/22-Smith”). This allows users to navigate and browse historical versions using the version number as a reference.

### Quality Control

DE quality control is primarily achieved through the audit process during registration. Currently, the audit process relies mainly on manual review, and it is evident shown in Table 5 that all 3 top-down repositories have established governance committees to conduct quality control audits. The audit process includes reviewing the basic attributes of elements (such as concepts and value domains), mapping or references between elements and controlled vocabularies, and the domain-specific expertise of elements. This audit process helps ensure the quality and authority of the published DEs, ensuring that their structural attributes are correct and appropriately specialized within their respective domains. However, it can be resource-intensive and time-consuming, requiring the involvement of experts. The bottom-up repositories MDM, on the other hand, cannot implement this process in the same way. Instead, it relies on repository administrators to conduct quality control audits. While this method can ensure only the basic structural integrity of data elements, its higher review efficiency makes it more suitable for bottom-up repositories handling large volumes of data element submissions.

A complete and well-defined registration workflow is a crucial part of quality control of DEs. MDM and CEDAR have not provided an entire registration process, while other repositories offer information on the registration workflow for DEs within the platform. They also assign identifiers for different registration statuses. METEOR and caDSR have more comprehensive registration statuses, with a finer-grained classification. In addition, only the NIH CDE Repository provides quality identifiers for DEs and includes only 1-level identifier (NIH-Endorsed). Other repositories do not appear to have detailed quality scoring or rating information. Only the NIH CDE Repository provides quality indicators for DEs, including a single-level indicator (NIH-Endorsed). Conversely, MDM relies on users to rate DEs, and other repositories do not seem to have detailed quality ratings or grading content.

**Table 5:** Analysis results of the 5 data element repositories in the quality control dimension, demonstrating the actions of each data element repository in data element quality control.

Repositories	Review method	Auditors	Quality mark	Registration				
				Registration workflow	Status identifier		Status type	Quality control records/documents
caDSR^a^	Manual review	Committee experts	No	Yes	Full life cycle	10		No
NIH^b^ CDE^c^ Repository	Manual review	Committee experts	NIH-Endorsed CDE	Yes	Full life cycle	2		No
METEOR^d^	Manual review	committee experts	No	Yes	Full life cycle	9		Yes
MDM^e^	Manual review	Portal administrator	No	N/A^f^	No	N/A		Partially provided
CEDAR^g^	N/A	N/A	No	N/A	No	N/A		No

^a^caDSR: Cancer Data Standards Registry and Repository.

^b^NIH: National Institutes of Health.

^c^CDE: Common Data Element.

^d^METEOR: Metadata Online Registry.

^e^MDM: Medical Data Model.

^f^N/A: not applicable.

^g^CEDAR: Center for Expanded Data Annotation and Retrieval.

### Semantic Annotation

The repositories achieve semantic annotation by standardizing the mapping of DEs to terminology systems, ensuring internal semantic consistency (Table 6). The primary terminology systems used by these repositories include Unified Medical Language System (UMLS), Logical Observation Identifiers Names and Codes (LOINC), and Systematized Nomenclature of Medicine—Clinical Terms (SNOMED CT), with others such as National Cancer Institute Thesaurus (NCIT) and National Center for Biomedical Ontology (NCBO) also being used. METEOR has developed its internal glossary and achieves semantic annotation through metadata items called “Glossary Items (GIs).” GIs share the same DE framework as other elements but store the definition of a term. Other DEs can achieve semantic annotation by referencing the corresponding GI associated with a specific term. Creating and referencing internal glossaries effectively harnesses the advantages of the ISO/IEC 11179 DE framework. GIs essentially facilitate clustering according to the DE framework, including object class, property, value domain, and more. DEs belonging to the same object class can be associated with the terminology item by referencing it. For instance, by querying the GI item “person,” you can observe all DEs that reference this term as their object class. This clustering enhances the interrelatedness of DEs at the conceptual level. However, the shortcomings of internal glossaries are also evident. If DEs need to be used across institutions, there is a need for remapping terminology, or semantic inconsistencies may persist. Regarding semantic interoperability, internal glossaries are less effective referencing internationally recognized terminology repositories.

**Table 6:** Analysis results of the 5 data element repositories in the semantic annotation dimension, presenting the measures taken by each data element repository to semantically standardize data elements.

Repositories	Annotation source	Mapping vocabulary	Granularity	Annotation method	Annotation content
caDSR^a^	Controlled vocabulary	NCIT^b^	DE^c^ concept and permissible value	Manual mapping	Terms and links
NIH^d^ CDE^e^ Repository	Controlled vocabulary	NCIT, UMLS^f^, etc	DE concept and permissible value	Manual mapping	Terms and coding
METEOR^g^	Self-built vocabulary	Self-built vocabulary	DE concept	Manual mapping	Terms and links
MDM^h^	Controlled vocabulary	UMLS, LOINC,^i^ and SNOMED CT^j^	DE concept and description	Automatic mapping	Terms and coding
CEDAR^k^	Controlled vocabulary	NCBO^l^	DE concept and permissible value	Manual mapping	Terms and links

^a^caDSR: Cancer Data Standards Registry and Repository.

^b^NCIT: National Cancer Institute Thesaurus.

^c^DE: Data Element.

^d^NIH: National Institutes of Health.

^e^CDE: Common Data Element.

^f^UMLS: Unified Medical Language System.

^g^METEOR: Metadata Online Registry.

^h^MDM: Medical Data Model.

^i^LOINC: Logical Observation Identifiers Names and Codes.

^j^SNOMED CT: Systematized Nomenclature of Medicine—Clinical Terms.

^k^CEDAR: Center for Expanded Data Annotation and Retrieval.

^l^NCBO: National Center for Biomedical Ontology.

### Service Support

A robust retrieval system can enhance the discoverability of data resources within repositories. Each of the 5 repositories possesses unique search capabilities, for instance, caDSR and NIH CDE Repository allow users to search by the names of NIH-affiliated institutions (Table 7). METEOR and MDM allow users to construct search queries using Boolean operators and keywords. Furthermore, these platforms also differ in their secondary filtering criteria, with caDSR and METEOR supporting additional filters such as submitting organization, registration status, and registering organization, among others.

The repositories offer personalized services to users, including features such as personal favorites in NIH CDE Repository and METEOR, enabling users to collect elements of interest and record and browse their own created or edited metadata. In CEDAR, DEs are organized in a folder structure, facilitating the categorization and management of metadata.

With regard to data element download and export services, all repositories except CEDAR offer support for multiple export formats. MDM supports export in 18 formats, including Comma Separated Values (CSVs) and Operational Data Model (ODM), but it is limited to exporting data by form and allows only 50 downloads per week. METEOR provides Word and PDF export formats with lower levels of structure, which can impact interoperability. caDSR and NIH CDE Repository allow DEs and forms to be exported in various structured document formats such as EXCEL, XML, and JSON, providing a relatively comprehensive download service. On the other hand, CEDAR offers only JSON source code for elements without direct download capabilities. Although it has a REST API interface, it may not be as convenient for nonbatch exports.

All 4 platforms except CEDAR provide web-based metadata comparison tools, but they differ in the dimensions they support for comparison. MDM and METEOR can perform horizontal comparisons for all information of 2 DEs, while caDSR supports comparisons for multiple DEs. NIH CDE Repository offers vertical comparisons, allowing users to compare DEs with their historical versions. In addition to the comparison tools, MDM also provides a rich set of auxiliary tools, including ODMedit (for creating ODM format DEs and forms) [57], CDEGenerator (for visualizing concept frequencies in forms) [58], OpenEDC (for web-based data collection using forms), and more. MDM offers a more significant number of tools and functionality than other repositories.

**Table 7:** Analysis results of the 5 data element repositories in the service dimension, mainly presenting the various services provided by each data element repository on its portal website to help users better use the repository and data elements.

Repositories	Features of retrieval	Results secondary screening	Register account	Account service	Sharing agreement	Download service	Download granularity	Export format	Comparison tool	Other tools
caDSR^a^	Abbreviation of the institute’s name, identifier, etc	Registration status, submitter, etc	N/A^b^	N/A	N/A	Unlimited downloads, batch download	DE and form	EXCEL, XML, and JSON	Compare 2 or more DEs^c^	Form creation
NIH^d^ CDE^e^ Repository	Abbreviation of the institute name, identifier, etc	Data type, submitter, etc	UTS^f^ account	Personal favorites, browsing history, etc	N/A	Unlimited downloads, batch download	DE and form	EXCEL, XML, JSON	Compare different versions of DE	Not support
METEOR	Keywords, identifier, Boolean operators, etc	Registration organization, data type, etc	Internal account	Personal favorites and settings, browsing history, etc	N/A	Unlimited downloads, batch download	DE	Word, PDF	Compare 2 DEs	DE creation
MDM^g^	Keywords, Boolean operators, wild card character, etc	Keywords, research field	Internal account	Personal favorites, browsing history, etc	Four version CC 4.0 licenses	50 forms per week	Form	CSV, EXCEL, SQL, and other 18 formats	Compare 2 or more DEs	Web-based date capture, visualization, visual analysis tools, etc
CEDAR^h^	Keywords, terminology, etc	Data type, version, etc	Internal account	API^i^ keys, personal folder	N/A	Not support	Not support	JSON code	Not support	DE and form creation

^a^caDSR: Cancer Data Standards Registry and Repository.

^b^N/A: not applicable.

^c^DEs: Data Elements.

^d^NIH: National Institutes of Health.

^e^CDE: Common Data Element.

^f^UTS: UMLS Terminology Services.

^g^MDM: Medical Data Model.

^h^CEDAR: Center for Expanded Data Annotation and Retrieval.

^i^API: Application Programming Interface.

### Usability

We analyze the usability of the repositories from 3 perspectives: openness, accessibility, and intelligibility. Openness focuses on the extent to which the repository’s resources and services are available for browsing and use. Among the 5 repositories in the study, access is typically restricted by requiring user accounts. Regarding data resources, caDSR, METEOR, and MDM provide unrestricted browsing access, including both forms and DEs. However, the NIH CDE Repository restricts viewing some semantic annotation content. Regarding services, MDM and CEDAR restrict auxiliary tools to logged-in users, including web-based creation and submission of DEs, among other features. In contrast, the DE creation and registration tools in the other 3 top-down repositories are not open to regular users. CEDAR requires registration for access to all services and resources, but it provides source code and technical documentation on GitHub. In summary, caDSR and METEOR exhibit the highest level of openness regarding resources and tools (Table 8).

Accessibility considers the types of accessible resources, the methods of accessibility, and the extent to which resources are accessible. There are primarily 2 ways to access repository resources: web downloads and application programming interface (API) interfaces. CEDAR does not provide web downloads and offers only JSON source code and an API interface. MDM requires user login for downloading forms and performing batch downloads, with a limit of 50 forms per week. In contrast, caDSR and NIH CDE Repository allow free downloads and batch exports of DEs without the need for login, making them relatively more accessible regarding resource availability.

Intelligibility focuses on the availability of supplementary information provided by the repositories and the complexity of constructing DEs. First, all 5 repositories offer user guide documents on their portals, which introduce basic information and operations. In addition, CEDAR and caDSR have Archive and Wiki web pages to provide further information and support. The repositories also pay attention to teaching concepts related to DEs. Since not all users have a computer-related background, all 4 platforms except MDM provide introductions or tutorials on metadata, DEs, and the ISO/IEC 11179 standard.

In addition, most repositories lack descriptions and visual representations of their data resources’ coverage areas and quantities. On its portal page, MDM provides visualizations of its DEs categorized by proportion, which can help users understand the resources within the repository. Compared with the complexity of DEs across the 5 platforms, MDM benefits from its self-built framework, resulting in relatively more straightforward and more concise DEs with better comprehensibility. In contrast, other platforms build their DEs based on the ISO/IEC 11179 standard and often expand or subdivide the framework, increasing the amount of information and complexity, which can affect comprehensibility.

**Table 8:** Analysis results of the 5 data element repositories in the usability dimension, mainly focusing on openness, accessibility, and usability, and comprehensively evaluating the usability of each data element repository.

Repositories	Openness										Accessibility									Intelligibility				
	Open access	Restriction	Create and submit		Open source		Auxiliary tool		Method			Limitation		Batch download		Quantity limitation		User guide			DE tutorial		DE complexity	
caDSR^a^	DEs^b^	No		No		No		Yes		Download and API^c^			No		Yes		No		Document			Yes		High
NIH^d^ CDE^e^ Repository	DEs and forms	Partial DEs		No		No		Yes		Download and API			No		Yes		No		Documents			Yes		High
METEOR^f^	DEs	No		No		No		Yes		Download			No		Require log-in		No		Document			Yes		Middle
MDM^g^	DEs and forms	No		Yes		No		Partially require log-in		Download			No		Require log-in		50 forms per week		Video			No		Low
CEDAR^h^	DEs and forms	All resources		Yes		Yes		Require log-in		JSON code and API			Require log-in		No		No		Video and document			Yes		Middle

^a^caDSR: Cancer Data Standards Registry and Repository.

^b^DEs: Data Elements.

^c^API: Application Programming Interface.

^d^NIH: National Institutes of Health.

^e^CDE: Common Data Elements.

^f^METEOR: Metadata Online Registry.

^g^MDM: Medical Data Model.

^h^CEDAR: Center for Expanded Data Annotation and Retrieval.

### Practice of FAIR Principles

Finally, this study supplemented the analysis by evaluating the extent to which the 5 repositories comply with the FAIR principles. The level of compliance was categorized into 4 groups: complies completely, complies entirely, fails to comply, and unclear. The detailed content of each principle in FAIR can be found in Multimedia Appendix 2.

**Figure 3:**
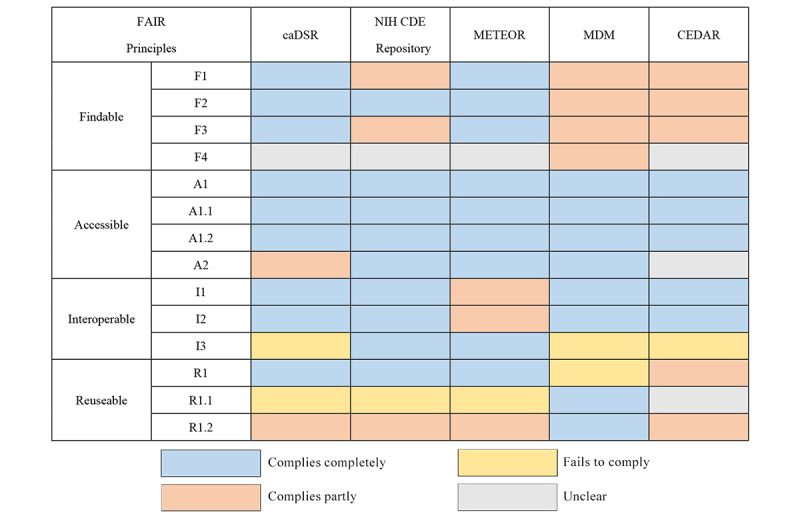
Visualization of FAIR (Findable, Accessible, Interoperable, and Reusable) practices in 5 repositories, with practices divided into 4 levels: complies completely, complies partly, fails to comply, and unclear. Detailed subprinciples of FAIR are shown in Multimedia Appendix 2. caDSR: Cancer Data Standards Registry and Repository; CDE: Common Data Element; CEDAR: Center for Expanded Data Annotation and Retrieval; MDM: medical data model; METEOR: metadata online registry; NIH: National Institutes of Health.

**Figure 4:**
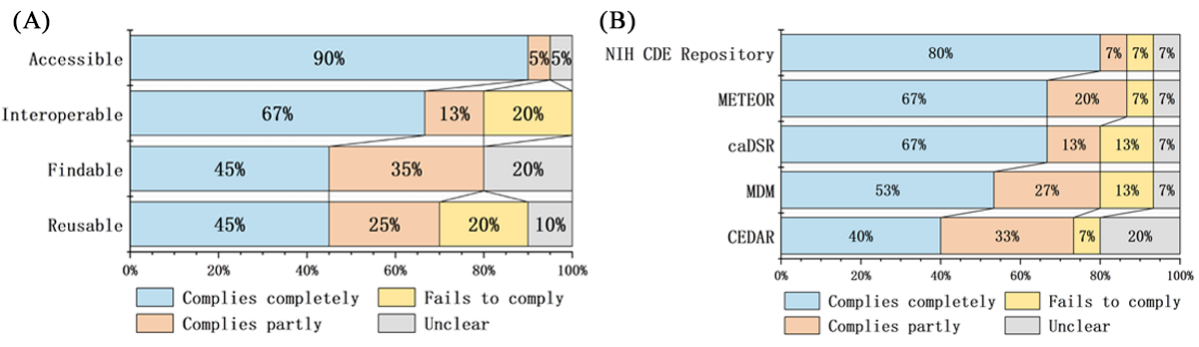
Statistics on the practice of FAIR (Findable, Accessible, Interoperable, and Reusable) principles based on Figure 3. (A) The figure starts from the perspective of FAIR principles and horizontally counts the practice of the 4 subprinciples in Figure 3. (B) The figure starts from the perspective of data element repositories and vertically counts the practice of FAIR principles in each repository in Figure 3. caDSR: Cancer Data Standards Registry and Repository; CDE: Common Data Element; CEDAR: Center for Expanded Data Annotation and Retrieval; MDM: medical data model; METEOR: metadata online registry; NIH: National Institutes of Health.

In Figure 3, a horizontal tally was conducted, with each of the 4 subprinciples of FAIR considered separately. The proportions of different levels of compliance to the subprinciples were calculated individually. For instance, considering the findable subprinciple, which comprises 4 principles (F1-F4), there are 20 cells. The proportions of “complies completely,” “complies partly,” “fails to comply,” and “unclear” were then calculated for these 20 cells. The same process was applied to the remaining 3 subprinciples. Based on this step, Figure 4A was generated, depicting the overall adherence of repositories to each subprinciple. Figure 4B, calculated using the same method on a column basis, illustrates each repository’s implementation of the FAIR principles.

In comparison, among the 5 repositories, the NIH CDE Repository demonstrates the highest level of compliance with the FAIR principles, while CEDAR falls behind the other 4 platforms. When examining the 4 subprinciples of FAIR, overall, Accessibility is relatively well practiced and at the same time, Findability and Reusability have lower percentages of full compliance, indicating subpar adherence to these principles’ aspects.

In the “Findable” principle, F4 “(Meta)data are registered or indexed in a searchable resource” is crucial for ensuring the discoverability of data resources on the web. Most of the 5 repositories analyzed in this study did not fully practice this aspect, impacting their web-based resources’ discoverability. Only MDM has been registered in the international academic domain of registry and indexing for repositories, such as re3data and FAIR sharing.

In terms of the “Specific (meta)data are referred to by their identifier” subprinciple of interoperability, 3 repositories did not fully comply. This is mainly due to a lack of rich cross-referencing between DEs. METEOR had the best compliance with this subprinciple, as it provides comprehensive reference information for DEs on their detail pages.

Regarding the practice of “Reusability,” the issues with the repositories primarily focus on data usage licenses and source information. Most repositories lack clear data usage licenses, which hinders data sharing. In addition, source information is often limited, with most repositories providing only submitter and time stamp information. Details about how the data were created and whether they had been previously published are typically not provided, impacting reusability.

## Discussion

### Principal Findings

The results of the analysis provide us with an overview of 5 DE repositories. The 2 approaches in repositories, namely, the top-down and bottom-up approaches, bring about differences and distinct characteristics regarding resources, semantics, and quality control. The community-driven, bottom-up approach where users submit resources, as seen in MDM and CEDAR, results in a richer pool of resources. This implies that repositories of this type need to implement more automation in various activities, including automated verification and terminology mapping. On the other hand, the top-down approach is the opposite of community-driven models. It relies on collaboration among experts from various domains. Expert committees are involved in designing, creating, and reviewing DEs in all 3 repositories following this approach. DEs following this approach have higher quality and authority, with a finer granularity in semantic annotations. However, consideration should still be given to their applicability outside the specific institution or research context. For example, DEs provided by repositories such as caDSR and NIH CDE Repository may be tailored to particular NIH-affiliated institutions and research scenarios. Conversely, community-driven DEs have a broader source base, potentially better reflecting real-world research situations. Their cross-study applicability might be more extensive.

Balancing the complexity and usability of DEs and repository metamodels is crucial. The data model structures built upon the ISO/IEC 11179 standard can be complex, and clinical researchers may not easily understand their underlying frameworks. It is essential to strike a balance to ensure that the repository remains user-friendly and accessible to its intended audience. Simplifying the framework, however, can complicate the organization of the repository. This may reduce the available information, which can negatively impact activities such as DE deduplication, establishing relationships, and hinder the development of advanced applications such as intelligent recommendations. While the self-built model of MDM is simple and user-friendly, it can organize resources only at the level of forms, lacking granularity down to the level of individual DEs. In contrast, repositories such as caDSR, built on the ISO/IEC 11179 standard, require more investment in learning and usage, but they offer more comprehensive and detailed management and organization capabilities.

### Standardize Data Sharing

Promoting data sharing does not necessarily mean unrestricted sharing. DE sharing also requires clear agreements and statements. Among the 5 repositories in this study, only MDM provides 4 different versions of the CC-4.0 license as options for form resources, which offers clarity in licensing for these resources. The other 4 repositories have not provided such information on their platforms, and their affiliated institutions’ data policies regarding the applicability to resources within these repositories are also somewhat unclear. Overall, these repositories seem to focus less on data sharing and reuse.

In the rapidly evolving landscape of open science, many mature examples of data-sharing strategies can serve as valuable references [59,60]. DEs are a form of data, and designing their sharing strategies can benefit from looking at the practices of other data-sharing platforms. We recommend that repositories clearly define protocols for sharing and reusing DEs in their portals. Furthermore, they should offer granularity down to the level of individual DEs, allowing resource submitters to choose specific sharing agreements. This approach can prevent unrestricted sharing and ensure greater control over DE access and usage.

### The Interconnected Ecosystem of Repositories

While DE repositories facilitate the integration of DEs across institutions and projects, the gaps between DE repositories should not become new barriers to integration. In this study, the 5 repositories analyzed do not support direct sharing and exchange of resources among each other. Instead, resources must be exported and then recreated in the target repository. However, the exported formats may not be highly structured, and there is no support for importing these files for quick creation in another repository.

Despite most repositories being built based on the ISO/IEC 11179 standard, there is still a lack of interoperability and data exchange between these repositories. These limitations suggest establishing a comprehensive interconnected ecosystem for DE repositories. Both top-down and bottom-up approaches can complement each other in achieving this goal, thereby avoiding redundant construction and facilitating domain-specific developments. This can ultimately lead to more efficient and collaborative medical research efforts.

To build the interconnected ecosystem of repositories, our recommendations are as follows:

- Choose standardized repository frameworks (such as the ISO/IEC 11179 standard) and terminology systems (eg, UMLS) to avoid the need for secondary mapping of underlying frameworks or semantics.
- Enhance the export of DEs to provide more structured documents, such as CSV and JSON.
- Develop DE creation features that offer rapid import services, supporting content creation from structured documents.
- Consider developing a unified interface, like QL4MDR [40].

### Enrich Information About DEs

A significant portion of DEs in DE repositories remains at the level of satisfying basic framework information. That is, they provide fundamental semantic information but lack application-oriented details. This includes contextual information such as applicable scenarios, background details, and application outcomes. In this scenario, DEs are isolated fragments scattered throughout the repository, providing users minimal application support. Users are left uncertain whether a DE adheres to a particular standard or belongs to a specific data set, making it challenging to select accurate DEs and organize them into the required format. The repository also falls short in delivering advanced services such as intelligent recommendations.

Therefore, this study suggests that DE repositories should enrich the application information of DEs to support their practical use. We categorize application information into two aspects: (1) Application scenarios and background details: specifying the scenarios for which DEs are applicable, whether generic or specialized, and the standards or data sets from which they originate. Such contextual information can assist the repository in better associating and organizing relevant DEs. (2) Performance-related information: this can include statistics on the number of applications of a DE and user ratings, feedback, and other relevant details.

Furthermore, we recommend that the repositories consider using ontology resources to provide standardized terminology. Mature ontology repositories and tool kits, such as NCBO BioPortal [61] and Ontology Lookup Service [62], offer a wealth of ontology resources and support the download and localization of various ontology resources or their invocation through APIs. By using methods such as precise matching and semantic similarity calculation, DEs can be mapped to ontology terms, thereby standardizing DE concepts, value domains, and so on. This can provide specific term annotations for DEs and further enrich the available information.

### Focus on Sensitive Data Protection

The existing repository contains DEs that collect sensitive information such as ID numbers, addresses, and phone numbers. However, these elements lack specific classification or identification to indicate that these DEs are used to collect sensitive data and may need to be deidentified or deleted. While the repository does not contain original research data, this remains a crucial issue for subsequent DE usage. We propose that the repository should align with the Health Insurance Portability and Accountability Act [63] or other relevant regulations and map the repository’s DEs with the protected personal health information. The repository should create classifications and identifiers for privacy-related DEs. This will serve as a reminder to users about the sensitivity of such data and promote standardized usage practices.

Addressing the balance between FAIR data-sharing principles and privacy protection, we emphasize that FAIR promotes secure, compliant, and interoperable data sharing, not unrestricted dissemination. It advocates for data classification and the application of tailored sharing environments. Privacy data can be deidentified or directly removed during the aggregation phase. In subsequent sharing and reuse processes, while adhering to FAIR principles, we should establish a secure usage environment and sharing guidelines for the data. This includes data classification and grading, implementing differential sharing protocols, and using privacy-enhancing technologies such as privacy computing and federated learning to control data accessibility. This approach ensures effective data sharing and reuse under the FAIR principles while upholding privacy protection.

### Implications

#### Theoretical Implications

In contrast to existing research that mainly concentrates developing specific technical aspects of DE repository construction, this study compares 5 typical DE repositories within the medical field and systematically evaluates and analyzes them. Furthermore, this study introduces a novel analysis framework consisting of 7 dimensions and 36 secondary indicators, based on the ISO/IEC 11179 standard and integrated with the FAIR principles. While this study focuses on the analysis of 5 DE repositories, we are confident that the proposed framework holds broad applicability to a wide range of repositories in the medical field. First, the 5 repositories included in this study have good representativeness, and their functions basically cover small repositories such as samply.MDR and CoMetaR. Therefore, the dimensions and indicators constructed by referring to these repositories can better cover general DE repositories and have more detailed content to be mined. Second, the ISO/IEC 11179 standard is an internationally used standard for the construction of DE repositories, and the FAIR principle is also a widely recognized data management and sharing guideline. Therefore, the dimensions and indicators constructed based on these 2 documents also have good applicability. Finally, in the process of constructing the analysis framework, we invited experts in data management and standardization to discuss and suggest the analysis framework. Simultaneously, the ISO/IEC 11179 standard provides specific definitions for the concept model of DEs and standardizes related management activities. Integrating of these 2 components in the analytical framework serves as the foundation for potential future research endeavors, allowing for further refinement of relevant standards and theories related to DE repositories.

#### Practical Implications

The practical significance of this study lies in its potential to drive the construction of DE repositories, facilitating a more robust implementation of the FAIR principles during the construction and management processes. This, in turn, contributes to a more substantial role in the data-driven advancement of medicine. For DE repository administrators, this study’s findings assist them in understanding the repository’s strengths and limitations, offering the necessary information for further improvements to the repository.

In addition, the integrated information on DE repositories from this research may hold practical implications for individuals involved in medical informatics research. For clinical research data managers, this information can assist them in gaining a better understanding of DE repositories. They can use this knowledge to make informed choices regarding suitable repositories and reuse DEs, reducing redundant design work in the clinical research process. For computer experts developing medical information systems, this research encompasses resource organization and management information from multiple repositories, along with service design offered by web apps. This can reference the top-level structure of DE repositories within their respective institutes.

### Conclusions and Limitations

Medical DEs enhance data quality, foster data reuse, and maximize the value of data in the era of health big data. They also form the foundational basis for AI-based medical systems. This study, using a constructed multidimensional analytical framework, evaluates and analyzes the current state of construction of typical medical DE repositories. It summarizes the characteristics of different repositories and provides recommendations based on identified issues. This study’s findings can promote the broader application of DE repositories, ensuring that DEs and repositories better serve clinical and medical research needs. Furthermore, this research can have applications in medical knowledge organization, and semantic representation, thus contributing to the development of AI technologies in medicine.

This study also has some limitations and areas for future improvement. First, the study had limited inclusion of databases, focusing solely on comprehensive, noncommercial DE repositories, all in the English language. Smaller or domain-specific repositories may have been overlooked. Furthermore, the data primarily came from repository websites and literature, with little attention given to other sources such as social media accounts. This approach might have missed some of the latest updates or changes. Therefore, future research will consider expanding the scope to include more repositories for analysis, relaxing constraints related to quantity and language. In addition, efforts will be made to enhance the generality of the analysis framework and develop a practical model for DE repositories.

## Appendix

Multimedia Appendix 1 Complete overview of the analysis framework.

Multimedia Appendix 2 Details of the FAIR principle.

## Abbreviations

AI: artificial intelligence
API: application programming interface
caDSR: Cancer Data Standards Registry and Repository
CDE: Common Data Element
CEDAR: Center for Expanded Data Annotation and Retrieval
CSV: Comma Separated Values
DE: data element
DSS: data set specification
FAIR: Findable, Accessible, Interoperable, and Reusable
GI: Glossary Item
ISO/IEC: International Organization for Standardization/International Electrotechnical Commission
LOINC: Logical Observation Identifiers Names and Codes
MDM-Portal: Portal of Medical Data Models
METEOR: Metadata Online Registry
NCBO: National Center for Biomedical Ontology
NCIT: National Cancer Institute Thesaurus
NIH: National Institutes of Health
NINDS: National Institute of Neurological Disorders and Stroke.
ODM: Operational Data Model
OWL: Web Ontology Language
SNOMED CT: Systematized Nomenclature of Medicine—Clinical Terms
UMLS: Unified Medical Language System
