# Supplementary material for: Toward Better Semantic Interoperability of Data Element Repositories in Medicine: Analysis Study
Source: JMIR Med Inform. 2024 Sep 30;12:e60293. doi: 10.2196/60293 (PMC11474123; doi:10.2196/60293)
Supplement: Multimedia Appendix 1 [file medinform_v12i1e60293_app1.docx]

**Abbreviations:** DE: Data Element

**Table S1** Description of Data Resource indicators.

| Indicator | Description |
| --- | --- |
| Area | Biomedical research fields covered by the data resource collection in the data element repository |
| Type | Type of data resources in the data element repository, primarily data elements, some including forms |
| Total Amount | Total amount of various data resources accumulated in the data element repository |
| Submitter | Primary submitters of data resources in the data element repository, excluding resources shared by other repositories. |

**Table S2.** Description of Resource organization indicators.

| Indicator | | Description |
| --- | --- | --- |
| Framework | | The underlying structural framework for constructing data elements in the data element repository, mostly using the data element framework from ISO 11179. |
| Naming Specification | | Whether the data elements in the repository have clear naming specifications. |
| Classification Scheme | | Whether the repository organizes data elements from multiple dimensions. |
| **Identifier** | | data element identifier. |
|  | Name | The name of the unique identifier of data element in the repository. |
|  | Encoding | The encoding rules for the unique identifier of data element in the repository. |
| **Traceability** | | Traceability of data element |
|  | Submitter/Source Information | Whether basic information of data element submitter or source is available. |
|  | Source Link | Whether there is a source link if data element is shared from other repositories. |
|  | Source Identifier | Whether the unique identifier of data element in the source system is provided. |
|  | Internal Citation link | Whether the internal citation of data element in the repository has a detailed page link. |
| **Version Control** | | The version control situation of data element in the repository. |
|  | Historical Versions Accessible | Whether the content of historical versions of DE is accessible. |
|  | Version Encoding | Whether historical version numbers are uniformly encoded. |

**Table S3.** Description of Quality control indicators.

| Indicator | | Description |
| --- | --- | --- |
| Review Method | | The review method when data element is submitted, such as manual review or other methods. |
| Auditors | | The personnel who review data element. |
| Quality Mark | | Whether data element has a quality status mark, such as high-quality data element. |
| **Registration** | | The registration process of data element in the repository. |
|  | Registration Workflow | Whether there is a complete and clear data element registration workflow. |
|  | Status Identifier | Whether each stage in the data element registration flow has a corresponding status identifier, such as registered, replaced, etc. |
|  | Status type | The number of types for data element registration status classification. |
| Quality Control Records/Documents | | Whether data element quality control records or documents are provided. |

**Table S4.** Description of Semantic annotation indicators.

| Indicators | Description |
| --- | --- |
| Annotation Source | The source of the terminology for semantic annotation of data elements, such as external controlled vocabularies or self-constructed vocabularies. |
| Mapping Vocabulary | The specific vocabulary name used for annotation. |
| Granularity | The granularity of semantic annotation, at the level of data element concepts, value domains, etc. |
| Annotation Method | The method of semantic annotation, such as automatic mapping by tools or manual addition of annotations. |
| Annotation Content | The specific content included in semantic annotation, such as the terms in the concept, unique identifiers in the source vocabulary, and source links, etc. |

**Table S5.** Description of Service support indicators.

| Indicators | Description |
| --- | --- |
| Features of Retrieval | Special retrieval functions provided by the repository portal, such as special retrieval terms, operators, etc. |
| Results Secondary Screening | The function of secondary screening of initial retrieval results according to different dimensions. |
| Register Account | The user account system of the repository portal. |
| Account Service | Personalized services provided by the repository for registered users, such as favorites, browsing history, etc. |
| Sharing Agreement | Whether the repository provides specific agreements or specifications for the sharing of data elements. |
| Download Service | The situation of data resource download services provided by the repository, including downloadable quantity, batch download, etc. |
| Download granularity | The levels at which the repository provides data resource download services. |
| Export Format | The formats available for data resources. |
| Comparison Tool | The data element comparison tool provided by the repository to assist in the comparison of related data elements. |
| Other Tools | Whether the repository provides other related tools to assist in the creation, analysis, and use of data elements. |

**Table S6.** Description of Usability indicators.

| Indicators | | Description |
| --- | --- | --- |
| **Openness** | | The degree of openness of data resources and services in the repository. |
|  | Open access | The types of data resources open in the repository. |
|  | restriction | Whether there are restrictions on browsing data resources. |
|  | create and submit | Whether the creation and submission services of data elements are open to individual users. |
|  | Open source | Whether the repository is open source. |
|  | auxiliary tool | Whether the repository's tool services are open. |
| **Accessibility** | | The degree of accessibility of data resources in the repository. |
|  | method | The method of obtaining data resources. |
|  | limitation | Whether there are restrictions on obtaining data resources. |
|  | Batch download | Whether batch acquisition services of data resources are provided. |
|  | Quantity limitation | Whether there are quantity limits on obtaining data resources. |
| **Intelligibility** | | Whether users can better understand and use data element resources. |
|  | User guide | Whether the repository provides a user guide. |
|  | DE Tutorial | Whether basic teaching content about data elements is provided. |
|  | DE Complexity | The complexity of data elements, mainly based on their structure, the amount of information provided, etc. |
