# Supplementary material for: Toward Better Semantic Interoperability of Data Element Repositories in Medicine: Analysis Study
Source: JMIR Med Inform. 2024 Sep 30;12:e60293. doi: 10.2196/60293 (PMC11474123; doi:10.2196/60293)
Supplement: Multimedia Appendix 2 [file medinform_v12i1e60293_app2.docx]

| FAIR principles |  |  |
| --- | --- | --- |
| Findable | F1 | (Meta)data are assigned a globally unique and persistent identifier. |
|  | F2 | Data are described with rich metadata (defined by R1 below). |
|  | F3 | Metadata clearly and explicitly include the identifier of the data they describe. |
|  | F4 | (Meta)data are registered or indexed in a searchable resource. |
| Accessible | A1 | (Meta)data are retrievable by their identifier using a standardized communications protocol. |
|  | A1.1 | The protocol is open, free, and universally implementable. |
|  | A1.2 | The protocol allows for an authentication and authorization procedure, where necessary. |
|  | A2 | Metadata are accessible, even when the data are no longer available. |
| Interoperable | I1 | (Meta)data use a formal, accessible, shared, and broadly applicable language for knowledge representation. |
|  | I2 | (Meta)data use vocabularies that follow FAIR principles. |
|  | I3 | (Meta)data include qualified references to other (meta)data. |
| Reusable | R1 | (Meta)data are richly described with a plurality of accurate and relevant attributes. |
|  | R1.1 | (Meta)data are released with a clear and accessible data usage license. |
|  | R1.2 | (Meta)data are associated with detailed provenance. |
|  | R1.3 | (Meta)data meet domain-relevant community standards. |
